# Impact of Nutritional Status on Neutrophil-to-Lymphocyte Ratio as a Predictor of Efficacy and Adverse Events of Immune Check-Point Inhibitors

**DOI:** 10.3390/cancers16101811

**Published:** 2024-05-09

**Authors:** Masahiko Sue, Yasuto Takeuchi, Shoichiro Hirata, Akinobu Takaki, Motoyuki Otsuka

**Affiliations:** 1 Graduate School of Medicine, Dentistry and Pharmaceutical Sciences, Okayama University, Okayama 700-8558, Japan; p58q0soo@okayama-u.ac.jp; 2 Department of Gastroenterology and Hepatology, Okayama University Hospital, Okayama 700-8558, Japan; p6q09uf6@okayama-u.ac.jp (S.H.); akitaka@md.okayama-u.ac.jp (A.T.); otsukamoto@okayama-u.ac.jp (M.O.); 3 Department of Regenerative Medicine, Center for Innovative Clinical Medicine, Okayama University Hospital, Okayama 700-8558, Japan

**Keywords:** immune-related adverse events, serum albumin, real-world practice

## Abstract

**Simple Summary:**

Immune checkpoint inhibitors have become widely used against malignant tumors. The neutrophil-to-lymphocyte ratio, an index focusing on immune cells, has been reported as a marker to predict the efficacy and side effects of anti-tumor drugs. In the present study, we found that the neutrophil-to-lymphocyte ratio shows an altered predictability of anti-tumor efficacy and side effects due to changes in nutritional status. Patients with carcinoma in situ are prone to changes in nutritional status depending on the progression of the cancer and the age of the patient. We believe that the results of this study are important findings in predicting the course of treatment for cancer patients.

**Abstract:**

The neutrophil -to-lymphocyte ratio (NLR) is useful for predicting the effectiveness of treatment with immune checkpoint inhibitors (ICIs) and immune-related adverse events (irAEs). Because a growing body of evidence has recently shown that the number of lymphocytes that comprise NLR fluctuates according to nutritional status, this study examined whether the usefulness of NLR varies in ICI treatment due to changes in nutritional status. A retrospective analysis was performed on 1234 patients who received ICI treatment for malignant tumors at our hospital. Progression-free survival (PFS) was significantly prolonged in patients with NLR < 4. Multivariate analysis revealed that the factors associated with the occurrence of irAE were NLR < 4 and the use of ipilimumab. However, when limited to cases with serum albumin levels <3.8 g/dL, lymphocyte counts significantly decreased, and the associations between NLR and PFS and between NLR and irAE occurrence disappeared. In contrast, when limited to the cases with serum albumin levels ≥3.8 g/dL, the associations remained, with significantly prolonged PFS and significantly increased irAE occurrence at NLR < 4. NLR may be a good predictive tool for PFS and irAE occurrence during ICI treatment when a good nutritional status is maintained.

## 1. Introduction

Immune checkpoint inhibitors (ICIs) are widely used to treat various tumors. ICIs have been shown to activate autoimmunity against tumors by blocking specific molecules [1]. Programmed cell death-1 (PD-1), programmed cell death ligand 1 (PD-L1), and cytotoxic T lymphocyte-associated antigen-4 (CTLA-4) are known to negatively regulate immune function and are targets of ICIs treatment [2]. While the high therapeutic efficacy of ICIs has been confirmed, serious immune-related adverse events (irAEs) have also been shown to occur [3,4]. irAEs are the side effect caused when this activated immune function is excessive, causing severe damage to various organs throughout the body [5,6,7,8,9]. However, this may contribute to favorable anti-tumor effects and a prolonged prognosis due to a strong immune response [10].

The status of immune cell activation has been reported to be inferred by laboratory tests such as blood counts [11]. For example, the neutrophil-to-lymphocyte ratio (NLR) is considered a useful indicator of systemic immune status during cancer treatment, and several studies have suggested that pretreatment NLR may be useful in predicting the development of irAEs and prognosis during ICI treatment [12,13,14,15,16].

In NLR, lymphocytes are particularly important, especially those associated with tumor immunity. These lymphocytes are known to fluctuate in number and function depending on nutritional status [17,18,19,20,21]. Many patients with carcinoma are often malnourished due to increased inflammatory cytokines associated with cancer, metabolic changes, and anorexia due to disease or systemic treatment [22]. Although this may affect lymphocyte-associated NLRs, there have been no reports examining the relationship between nutritional status and NLRs. In this study, we focused on serum albumin levels, a good indicator of nutritional status [23], to determine the impact of nutritional status on the usefulness of NLR toward predicting irAE occurrence and prognosis during treatment with ICIs.

## 2. Materials and Methods

### 2.1. Patients

This was a single-center, retrospective, observational study. We enrolled 1234 patients who received ICI treatment at our hospital between January 2016 and December 2022. Patients who were difficult to assess due to short observation periods or missing biochemical data at the start of treatment; who were difficult to follow-up due to hospital transfer or relocation; or who refused to consent to the study were excluded. The most common type of cancer was lung (*n* = 394, 31.9%), followed by gastrointestinal tract (*n* = 218, 17.6%), head and neck (*n* = 164, 13.3%), kidney (*n* = 163, 13.2%), melanoma (*n* = 138, 11.8%), liver (*n* = 68, 5.5%), genital (*n* = 65, 5.3%), and others (*n* = 24, 1.9%).

### 2.2. ICIs Treatment Regimens

The patients were treated with the following ICIs: (1) anti-PD-1 nivolumab, (2) anti-PD-1 pembrolizumab, (3) anti-CTLA-4 ipilimumab, (4) anti-PD-L1 atezolizumab, (5) anti-PD-L1 durvalumab, (6) anti-PD-L1 avelumab, and (7) a combination of anti-PD-1 nivolumab and anti-CTLA-4 ipilimumab. For each carcinoma, an ICI treatment regimen recommended by the guidelines and approved in Japan was initiated based on the judgment of the oncologists. Patients received ICI treatment until confirmation of disease progression, a drop in performance status to 2 or higher, or until unacceptable toxicity. When physicians decided to discontinue ICI treatment, patients received other chemotherapies or transition to the best supportive care.

### 2.3. Follow-up

Tumors were evaluated using computed tomography or magnetic resonance imaging at every period, according to the standard protocols for the treatment of each carcinoma. Responses were graded as complete response, partial response, stable disease, or progressive disease using the Response Evaluation Criteria in Solid Tumors 1.1 guidelines. The Common Terminology Criteria for Adverse Events 5.0 were used to assess irAEs.

### 2.4. Statistical Analyses

Baseline characteristics are presented as medians and ranges. Continuous variables were compared using the Wilcoxon rank-sum test, and for categorical values the chi-square test was employed. A Cox proportional hazards model was used to identify the factors associated with progression-free survival (PFS). Multivariate logistic regression analysis was performed to identify factors associated with irAE occurrence. Items with *p* < 0.1 in the univariate analysis were selected as adjustment factors in the multivariate analysis. The median value was used as the cutoff value for each item. Overall survival (OS) and PFS were estimated using the Kaplan–Meier method and compared among patient groups using the log-rank test. All significance tests were two-sided, and statistical significance was set at *p* < 0.05. Receiver operating characteristic curve analyses for the onset of irAEs were used to identify the cutoff values for NLR. The cutoff value used for serum albumin was 3.8 g/dL, as this was the median value. All analyses were performed using JMP software (version 16.0; SAS Institute Japan Ltd., Tokyo, Japan). 

## 3. Results

### 3.1. Patient Characteristics

The characteristics of the patients enrolled in the study at ICI treatment initiation are shown in Table 1. The median age of the included patients was 69 years, and 892 (72.3%) were male. The median serum albumin level was 3.8 g/dL. The median lymphocyte and neutrophil counts were 1172 and 3286/μL, respectively. The median NLR was 2.9. For each carcinoma, an ICI treatment regimen recommended by the guidelines and approved in Japan was initiated based on the judgment of the oncologists (Supplementary Table S1). The incidence of irAEs was 27.0% of all cases, with liver injury being the most common, accounting for 20.8% (Table 2). The median time to irAE occurrence was 68 days from the initiation of ICI treatment, and the median observation period was 280 days.

### 3.2. Factors Associated with ICI Response and irAE Occurrence

Based on the Cox proportional hazards model, Table 3 shows the analysis of factors related to PFS when using the ICI treatment. The univariate analysis showed that serum creatinine levels <0.8 mg/dL, NLR < 4, and the occurrence of irAEs were significantly associated with prolonged PFS. As per the multivariate analysis, NLR < 4 (odds ratio [OR] = 1.2, 95% confidence interval [CI] = 1.1–1.4; *p* = 0.033) and the occurrence of irAEs (OR = 1.9, 95% CI = 1.6–2.2; *p* < 0.01) were significant and independent determinants of prolonged PFS. 

Table 4 shows the analysis of predictive factors for irAE occurrence using logistic regression. The univariate analysis revealed that NLR < 4 and ipilimumab use were significant factors associated with the occurrence of irAEs. A multivariate analysis also showed that NLR < 4 (OR = 1.4, 95% CI = 1.0–1.9; *p* = 0.022) and the combination use of ipilimumab (OR = 3.6, 95% CI = 2.2–5.9; *p* < 0.01) were significant and independent determinants of irAE occurrence.

### 3.3. Serum Albumin Levels May Affect the Relationships between NLR and irAE Occurrence and between NLR and Therapeutic Effects of ICIs

**Figure 1:**
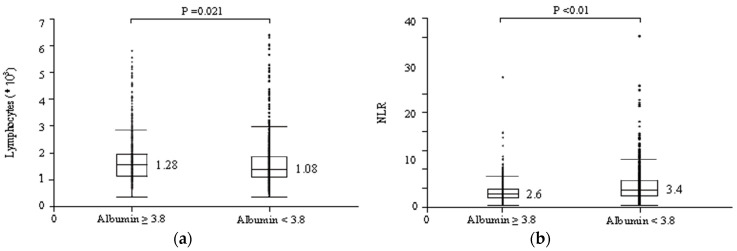
Changes in the number of lymphocytes and the NLR value according to the albumin values. (**a**) Comparison of lymphocyte number in cases with serum albumin levels ≥3.8 g/dL and <3.8 g/dL at the start of ICI treatment. The number of lymphocytes in patients with albumin ≥ 3.8 g/dL is higher than in patients with serum albumin levels <3.8 g/dL (*p* = 0.021). (**b**) Comparison of NLR value in cases with serum albumin levels ≥3.8 g/dL and <3.8 g/dL at the start of ICI treatment. NLR is significantly lower in patients with serum albumin levels ≥3.8 g/dL compared to those with serum albumin levels <3.8 g/dL (*p* < 0.01).

**Figure 2:**
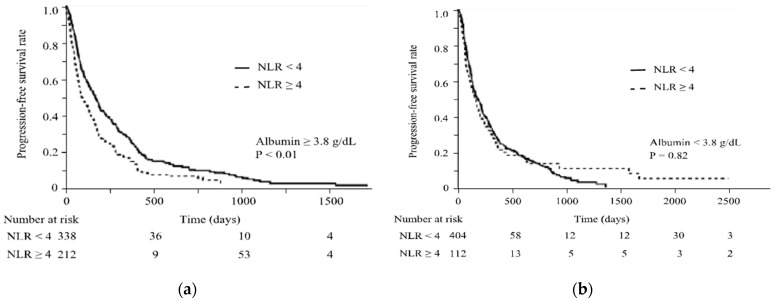
Progression-free survival according to the NLR status. Comparison of the survival curves in patients with NLR < 4 or with NLR ≥ 4 for PFS, in cases with serum albumin levels ≥3.8 g/dL (**a**) and <3.8 g/dL (**b**). While in cases with serum albumin levels ≥3.8 g/dL, PFS is significantly prolonged in patients with NLR < 4 (*p* < 0.01) (**a**), there is no significant difference in PFS according to NLR levels in cases with serum albumin levels <3.8 g/dL (**b**).

It was shown that albumin levels could alter the predictive ability of NLR for treatment response. The correlation coefficient between serum albumin levels and lymphocyte counts was 0.1257 (95% CI = 0.0679–0.1805). Hence, serum albumin levels and lymphocyte counts correlated with significant relevance (*p* < 0.01). As shown in Figure 1, the number of lymphocytes in patients with serum albumin levels ≥3.8 g/dL was predominantly higher than in patients with serum albumin levels <3.8 g/dL (1280 vs. 1080/μL, respectively; *p* = 0.021) (Figure 1a), and NLR was conversely significantly lower in patients with serum albumin levels ≥3.8 g/dL compared to those with serum albumin levels <3.8 g/dL (2.6 vs. 3.4, *p* < 0.01) (Figure 1b). Table 5 shows the results of the analyses of the predictive factors for irAE occurrence using logistic regression, depending on serum albumin levels. Further, a multivariate analysis showed that NLR < 4 (OR = 1.7, 95% CI = 1.1–2.6; *p* = 0.030) and the use of ipilimumab (OR = 4.0, 95% CI = 1.9–8.7; *p* < 0.01) were significant and independent determinants of irAE occurrence in patients with serum albumin levels ≥3.8 g/dL. In contrast, in patients with serum albumin levels <3.8 g/dL, the use of ipilimumab (OR = 3.3, 95% CI = 1.7–6.4; *p* < 0.01) was the only significant and independent determinant of irAE occurrence. As shown in Figure 2, patients with serum albumin levels ≥3.8 g/dL had significantly prolonged PFS when NLR < 4 (median survival, 5.6 vs. 3.1 months, *p* < 0.01; Figure 2a), while patients with serum albumin levels <3.8 g/dL showed no difference in PFS in terms of different NLR levels (median survival, 6.6 vs. 5.9, *p* = 0.82; Figure 2b). It was shown that albumin levels could alter the predictive ability of NLR for treatment response.

### 3.4. Effect of Albumin Levels on Overall Survival by in ICI Treatment

**Figure 3:**
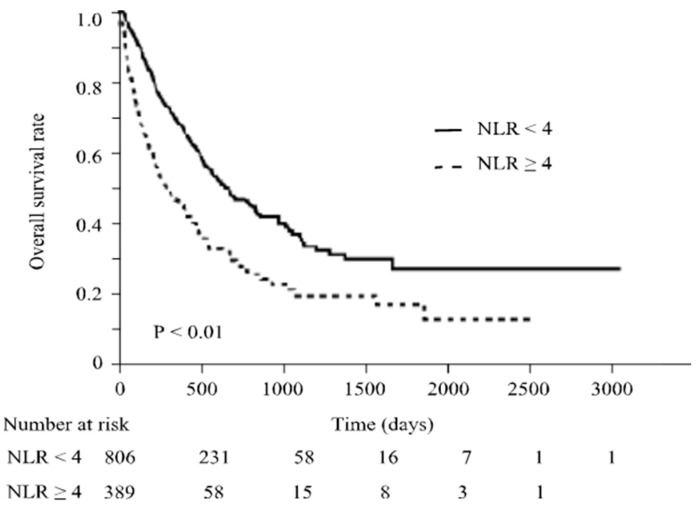
Overall survival according to the status of NLR value. OS in patients with different NLR values. OS was significantly prolonged in patients with NLR < 4 (*p* < 0.01).

**Figure 4:**
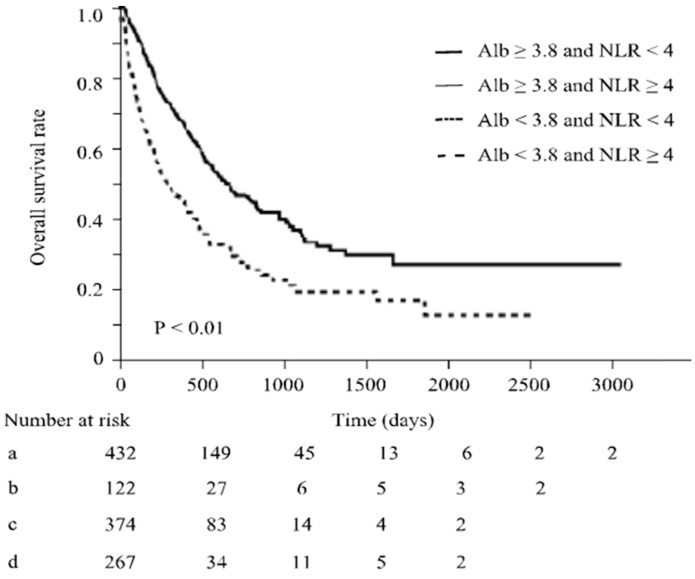
In patients with serum albumin levels >3.8 g/dL, OS was prolonged by approximately 33.1 months when NLR was <4. However, in patients with serum albumin levels <3.8 g/dL, OS was prolonged by approximately 16.2 months when NLR was <4. The duration of prognostic prolongation predicted by NLR was significantly shorter (*p* < 0.01) in cases with serum albumin levels <3.8 g/dL.

Regarding overall survival, when all patients were considered, it was significantly prolonged in patients with NLR < 4 (median survival, 21.9 vs. 10.0 months; *p* < 0.01) (Figure 3). Even in patients with NLR < 4, OS was most prolonged with serum albumin levels ≥3.8 g/dL. When serum albumin levels were <3.8 g/dL, although OS was also prolonged in cases with NLR < 4, its prolonged duration was significantly shorter than the cases with serum albumin levels ≥3.8 g/dL (median survival, 33.1 vs. 16.2 months, *p* < 0.01; Figure 4). These results suggest that decreased serum albumin levels also reduce the significance of NLR as a predictor of OS in ICI treatment.

## 4. Discussion

ICIs are important agents for the immunotherapy of malignant tumors. In this study, we examined, for the first time, the influence of nutritional status on NLR, which has been reported as a good predictor of treatment response and the development of irAEs during ICI treatment. Consistent with other reports [12,13,14,16], a low NLR at the start of ICI treatment is a significant predictor of irAE occurrence and improved treatment efficacy. However, when we focused on serum albumin levels, one of the indicators of the patients’ nutritional status, NLR was not a good predictor of irAE occurrence when serum albumin levels were low and was not useful for predicting the changes in PFS. 

Albumin is involved in protein metabolism. Other than a low nutritional status, albumin levels can also be decreased by other conditions such as nephrotic syndrome, impaired liver function, and inflammatory diseases [24,25,26]. However, the patients analyzed in this study had preserved performance status that allowed the induction of systemic chemotherapy, and there were no differences in albumin levels based on age. In addition, there were no comorbidities that could cause a decrease in albumin, such as nephrotic syndrome, persistent inflammatory disease, trauma, or hemorrhage. Even in patients with hepatocellular carcinoma, liver function was good and albumin levels were not decreased.

Cellular immunity is reduced under low-nutrient conditions. The fact that a decrease in cellular immunity leads to the atrophy of lymphoid tissues such as the thymus, lymph nodes, and spleen; a decrease in the number of peripheral blood lymphocytes; and a decrease in T lymphocytes and their functions is well documented [18,19,21,27]. Lymphocytes are important cells in the treatment of ICIs because ICIs bind to immune checkpoint molecules or their ligands and inhibit the transmission of immunosuppressive signals, thereby releasing the suppression of T-cell activation by immune checkpoint molecules and promoting T-cell antitumor effects [28,29,30]. NLR is considered an indicator that reflects the power of anti-tumor immunity or excessive immune response of these lymphocytes and has been used to investigate its association with irAE development and prognosis [31,32]. In this study, a positive correlation between serum albumin levels and lymphocyte counts was confirmed. It has been suggested that ICI activates lymphocytes in the priming and effector phases, thereby decreasing NLR by increasing lymphocytes in the affiliated lymph nodes and in the tumor. It has also been suggested that the effect of ICIs is greater when lymphocyte counts are high and NLR values are low before treatment begins. Therefore, although lymphocyte status may be an important predictor of response in ICI treatment, a positive correlation between serum albumin levels and lymphocyte counts was confirmed in this study. A lower serum albumin level was associated with a significantly lower lymphocyte count, and thus NLR, theoretically, increased with poor nutritional status. In addition, we revealed that changes in serum albumin levels were associated with changes in the occurrence of irAEs and NLR and between the therapeutic effect of ICIs and NLR. Because NLR is a tool for assessing nutritional status based on blood cell balance [33], when serum albumin levels are low, that is, when patients are undernourished, NLR is no longer able to accurately predict the occurrence of irAEs and the efficacy of ICIs.

The association between irAE occurrence and prolonged OS and the association with lower NLR have been reported [32]; however, it may not be appropriate to make predictions based on irAEs and NLR alone, since these variables change depending on subsequent treatment after treatment discontinuation due to irAEs or tumor progression, as well as on the general condition of the patient. Although it was confirmed that OS worsened when the serum albumin level was low, even with a low NLR, it was suggested that the usefulness of NLR for OS may change depending on nutritional status, while the possibility that worsening nutritional status itself may affect prognosis cannot be ruled out, making it difficult to accurately assess NLR in OS based on nutritional status. Considering that OS is not only affected by the therapeutic effect of ICI treatment but also by post-treatment is essential.

The present study has some limitations. First, there was heterogeneity and variability in the patient population, clinical setting, comorbidities, organ function, treatment duration, details of prior therapy, and follow-up time because this was a retrospective study using real clinical data. Secondly, in some cases, some biochemical data could not be collected. Third, it was difficult to ascertain the irAE grade. Fourth, the study was conducted at a single center with possible regional influences. Nevertheless, the identification of predictive factors is possible in the treatment of ICIs. NLR is one of the possible predictors of these. However, careful monitoring is required, especially for patients who are undernourished because of increased NLR due to lower lymphocyte counts. Furthermore, it remains to be determined whether the prediction of the frequency of irAEs and therapeutic efficacy using NLR becomes effective once the nutritional status is improved by means of therapeutic intervention.

## 5. Conclusions

In treatment with ICIs, NLR has so far been reported to be a useful tool for predicting the occurrence and prognosis of irAEs. However, its usefulness may be reduced if a good nutritional status is not maintained. NLR may be useful only when albumin levels, an indicator of nutritional quality, are high.

## Figures and Tables

**Table 1:** Characteristics of the patients receiving ICI treatment.

Patient Characteristics	Study Patients (*n* = 1234)
Age (years)	69 (60–95) ^+^
Gender	
Male (%)	892 (72.3)
Female (%)	342 (27.7)
White blood cells (/μL)	5325 (4150–6960) ^+^
Neutrophils (/μL)	3286 (2097–4610) ^+^
Lymphocytes (/μL)	1172 (813–1713) ^+^
NLR	2.9 (1.8–4.6) ^+^
Albumin (g/dL)	3.8 (3.3–4.0) ^+^
Total Bilirubin (mg/dL)	0.5 (0.4–0.7) ^+^
Aspartate aminotransferase (U/L)	20 (17–27) ^+^
Alanine aminotransferase (U/L)	15 (11–23) ^+^
Creatinine (mg/dL)	0.8 (0.7–1.0) ^+^
Cholinesterase (U/L)	246 (185–305) ^+^
Total cholesterol (mg/dL)	178 (151–210) ^+^
Immunoglobulin G (mg/dL)	1144 (953–1403) ^+^
Drug	
Nivolumab (%)	526 (42.6)
Pembrolizumab (%)	369 (29.9)
Atezolizumab (%)	187 (12.2)
Nivolumab + Ipilimumab (%)	76 (6.2)
Durvalumab (%)	47 (3.8)
Ipilimumab (%)	16 (1.3)
Avelumab (%)	13 (1.0)
irAE occurrence (%)	333 (27.0)
irAE grade (1 /2 /3 /4)	55 /150 /108 /19
Time to irAE occurrence (days)	68 (26–167) ^+^
Observation period (days)	280 (138–489) ^+^

^+^ Data are presented as the median (range); NLR, neutrophil-to-lymphocyte ratio; irAEs, immune-related adverse events.

**Table 2:** Types of irAEs.

Type of irAEs	All Events (*n* = 413)
Liver injury (%)	84 (20.8)
Pneumonia (%)	71 (17.2)
Thyroid dysfunction (%)	63 (15.3)
Rash (%)	54 (13.1)
Adrenocortical insufficiency (%)	28 (6.8)
Colitis (%)	29 (7.0)
Renal dysfunction (%)	16 (3.9)
Arthritis (%)	9 (2.2)
Cytopenia (%)	8 (1.9)
Pituitary dysfunction (%)	7 (1.7)
Infusion reaction (%)	7 (1.7)
Gastritis (%)	5 (1.2)
Type 1 diabetes (%)	5 (1.2)
Muscle pain (%)	3 (0.7)
Cholangitis (%)	3 (0.7)
Pancreatitis (%)	3 (0.7)
Others (%)	18 (4.4)

**Table 3:** Cox proportional hazards model of parameters associated with progression-free survival.

Parameters	Univariate Analysis		Multivariate Analysis	
	Hazard Ratio (Range) ^+^	*p*-Value	Hazard Ratio (Range) ^+^	*p*-Value
Age (≥70 years)	0.9 (0.83–1.1) ^+^	NS		
Sex (male)	0.9 (0.79–1.1) ^+^	NS		
NLR < 4	1.3 (1.1–1.5) ^+^	<0.01	1.2 (1.1–1.4) ^+^	0.033
Total Bilirubin (≥1.0 mg/dL)	1.0 (0.77–1.3) ^+^	NS		
Creatinine (<0.8 mg/dL)	1.2 (0.99–1.3) ^+^	0.056	1.1 (0.98–1.3) ^+^	NS
Immunoglobulin G (≥1200 mg/dL)	0.93 (0.74–1.2) ^+^	NS		
Fever within 24 h (present)	1.1 (0.79–1.3) ^+^	NS		
irAEs (present)	1.9 (1.6–2.2) ^+^	<0.01	1.9 (1.6–2.2) ^+^	<0.01

^+^ 95% confidence interval. NLR, neutrophil-to-lymphocyte ratio; irAEs, immune-related adverse events; NS, not significant.

**Table 4:** Logistic regression analysis of predictive factors for irAEs.

Parameters	Univariate Analysis		Multivariate Analysis	
	Odds Ratio (Range) ^+^	*p*-Value	Odds Ratio (Range) ^+^	*p*-Value
Age (≥70years)	1.1 (0.85-1.4) ^+^	NS		
Sex (male)	1.0 (0.78–1.3) ^+^	NS		
NLR < 4	1.4 (1.0–1.8) ^+^	0.042	1.4 (1.0–1.9) ^+^	0.022
Total Bilirubin (≥1.0 mg/dL)	0.8 (0.49–1.3) ^+^	NS		
Creatinine (≥0.8 mg/dL)	1.2 (0.90–1.5) ^+^	NS		
Immunoglobulin G (≥1200 mg/dL)	0.88 (0.58–1.3) ^+^	NS		
Ipilimumab (present)	3.7 (2.3–5.9) ^+^	<0.01	3.6 (2.2–5.9) ^+^	<0.01

^+^ 95% confidence interval; NLR, neutrophil-to-lymphocyte ratio; NS, not significant.

**Table 5:** Logistic regression analysis of factors related to irAE occurrence at different albumin levels.

	Albumin < 3.8 g/dL		Albumin ≥ 3.8 g/dL	
Parameters	Multivariate Analysis		Multivariate Analysis	
	Odds Ratio (Range) ^+^	*p*-Value	Odds Ratio (Range) ^+^	*p*-Value
NLR < 4	1.2 (0.74–1.8) ^+^	NS	1.7 (1.1–2.6) ^+^	0.030
Ipilimumab (present)	3.3 (1.7–6.4) ^+^	<0.01	4.0 (1.9–8.7) ^+^	<0.01

^+^ 95% confidence interval; NLR, neutrophil-to-lymphocyte ratio; NS, not significant.

## Data Availability

Due to the nature of this research, participants of this study did not agree for their data to be shared publicly, so supporting data are not available.

## Supplementary Materials

The following supporting information can be downloaded at: https://www.mdpi.com/article/10.3390/cancers16101811/s1, Table S1: Treatment regimens for each cancer.

## References

[1] Ribas A., Wolchok J.D. (2018). Cancer immunotherapy using checkpoint blockade. Science.

[2] Postow M.A., Callahan M.K., Wolchok J.D. (2015). Immune checkpoint blockade in cancer therapy. J. Clin. Oncol..

[3] Kumar P., Shikha S., Bellur P. (2020). Cancer immunotherapy with checkpoint inhibitor can cause autoimmune adverse events due to loss of Treg homeostasis. Semin. Cancer Biol..

[4] Friedman C.F., Proverbs-Singh T.A., Postow M.A. (2016). Treatment of the immune-related adverse effects of immune checkpoint inhibitors: A review. JAMA Oncol..

[5] Postow M.A., Sidlow R., Hellmann M.D. (2018). Immune-related adverse events associated with immune checkpoint blockade. N. Engl. J. Med..

[6] Fessas P., Possamai L.A., Clark J., Daniels E., Gudd C., Mullish B.H., Alexander J.L., Pinato D.J. (2020). Immunotoxicity from checkpoint inhibitor therapy: Clinical features and underlying mechanisms. Immunology.

[7] Myers G. (2018). Immune-related adverse events of immune checkpoint inhibitors: A brief review. Curr. Oncol..

[8] Wang D.Y., Salem J.E., Cohen J.V., Chandra S., Menzer C., Ye F., Zhao S., Das S., Beckermann K.E., Ha L. (2018). Fatal toxic effects associated with immune checkpoint inhibitors. A systematic review and meta-analysis. JAMA Oncol..

[9] Hoffner B., Rubin K.M. (2019). Meeting the challenge of immune-related adverse events with optimized telephone triage and dedicated oncology acute care. J. Adv. Pract. Oncol..

[10] Zhou X., Yao Z., Yang H., Liang N., Zhang X., Zhang F. (2020). Are immune-related adverse events associated with the efficacy of immune checkpoint inhibitors in patients with cancer? A systematic review and meta-analysis. BMC Med..

[11] Hopkins A.M., Rowland A., Kichenadasse G., Wiese M.D., Gurney H., McKinnon R.A., Karapetis C.S., Sorich M.J. (2017). Predicting response and toxicity to immune checkpoint inhibitors using routinely available blood and clinical markers. Br. J. Cancer.

[12] Diem S., Schmid S., Krapf M., Flatz L., Born D., Jochum W., Templeton A.J., Früh M. (2017). Neutrophil-to-lymphocyte ratio (NLR) and platelet-to-lymphocyte ratio (PLR) as prognostic markers in patients with non-small cell lung cancer (NSCLC) treated with nivolumab. Lung Cancer.

[13] Capone M., Giannarelli D., Mallardo D., Madonna G., Festino L., Grimaldi A.M., Vanella V., Simeone E., Paone M., Palmieri G. (2018). Baseline neutrophil-to-lymphocyte ratio (NLR) and derived NLR could predict overall survival in patients with advanced melanoma treated with nivolumab. J. Immunother. Cancer.

[14] Bagley S.J., Kothari S., Aggarwal C., Bauml J.M., Alley E.W., Evans T.L., Kosteva J.A., Ciunci C.A., Gabriel P.E., Thompson J.C. (2017). Pretreatment neutrophil-to-lymphocyte ratio as a marker of outcomes in nivolumab-treated patients with advanced non-small-cell lung cancer. Lung Cancer.

[15] Daly L.E., Power D.G., O’Reilly Á., Donnellan P., Cushen S.J., O’Sullivan K., Twomey M., Woodlock D.P., Redmond H.P., Ryan A.M. (2017). The impact of body composition parameters on ipilimumab toxicity and survival in patients with metastatic melanoma. Br. J. Cancer.

[16] Diehl A., Yarchoan M., Hopkins A., Jaffee E., Grossman S.A. (2017). Relationships between lymphocyte counts and treatment-related toxicities and clinical responses in patients with solid tumors treated with PD-1 checkpoint inhibitors. Oncotarget.

[17] Wolfson M., Strong C.J., Minturn D., Gray D.K., Kopple J.D. (1984). Nutritional status and lymphocyte function in maintenance hemodialysis patients. Am. J. Clin. Nutr..

[18] Fock R.A., Blatt S.L., Beutler B., Pereira J., Tsujita M., de Barros F.E., Borelli P. (2010). Study of lymphocyte subpopulations in bone marrow in a model of protein–energy malnutrition. Nutrition.

[19] Veldhoen M., Ferreira C. (2015). Influence of nutrient-derived metabolites on lymphocyte immunity. Nat. Med..

[20] Björkholm M., Bark S., Backman L., Jarstrand C., Holm G. (1993). Lymphocyte and granulocyte function in nutritionally depleted patients. The effect of 2 weeks of total parenteral nutrition. Clin. Nutr..

[21] Chandra R.K. (1977). Lymphocyte Subpopulations in Human Malnutrition: Cytotoxic and Suppressor Cells. Pediatrics.

[22] Barreira J.V. (2021). The Role of Nutrition in Cancer Patients. Nutr. Cancer.

[23] Lin Y.T., Lin P.T., Lin C.C., Wu T.H., Liu L.T., Su C.W., Teng W., Tsai C.Y., Huang C.H., Chen W.T. (2023). Adding nutritional status to the original BCLC stage improves mortality prediction for hepatocellular carcinoma patients in HBV-endemic regions. Am. J. Cancer Res..

[24] Kidney Disease Improving Global Outcomes (KDIGO) Glomerulonephritis Work Group (2012). KDIGO Clinical Practice Guideline for Glomerulonephritis. Kidney Int. Suppl..

[25] Bernardi M., Angeli P., Claria J., Moreau R., Gines P., Jalan R., Caraceni P., Fernandez J., Gerbes A.L., O’Brien A.J. (2020). Albumin in decompensated cirrhosis: New concepts and perspectives. Gut.

[26] Gabay C., Kushner I. (1999). Acute-phase proteins and other systemic responses to inflammation. N. Engl. J. Med..

[27] Chandra R.K. (1983). Numerical and functional deficiency in T helper cells in protein energy malnutrition. Clin. Exp. Immunol..

[28] Malas S., Harrasser M., Lacy K.E., Karagiannis S.N. (2014). Antibody therapies for melanoma: New and emerging opportunities to activate immunity (Review). Oncol. Rep..

[29] Vasaturo A., Di Blasio S., Peeters D.G., de Koning C.C., de Vries J.M., Figdor C.G., Hato S.V. (2013). Clinical Implications of Co-Inhibitory Molecule Expression in the Tumor Microenvironment for DC Vaccination: A Game of Stop and Go. Front. Immunol..

[30] Chen D.S., Mellman I. (2013). Oncology meets immunology: The cancer-immunity cycle. Immunity.

[31] Lee P.Y., Oen KQ X., Lim GR S., Hartono J.L., Muthiah M., Huang D.Q., Teo FS W., Li A.Y., Mak A., Chandran N.S. (2021). Neutrophil-to-Lymphocyte Ratio Predicts Development of Immune-Related Adverse Events and Outcomes from Immune Checkpoint Blockade: A Case-Control Study. Cancers.

[32] Michailidou D., Khaki A.R., Morelli M.P., Diamantopoulos L., Singh N. (2021). Association of blood biomarkers and autoimmunity with immune related adverse events in patients with cancer treated with immune checkpoint inhibitors. Sci. Rep..

[33] Yoshinaga O., Yumiko S., McMillan D.C., Miki C. (2017). Clinical burden of nutrition assessment in treatment for gastrointestinal cancer. J. Jpn. Soc. Parenter. Enter. Nutr..

