# Supplementary material for: Impact of Nutritional Status on Neutrophil-to-Lymphocyte Ratio as a Predictor of Efficacy and Adverse Events of Immune Check-Point Inhibitors
Source: Cancers (Basel). 2024 May 9;16(10):1811. doi: 10.3390/cancers16101811 (PMC11120021; doi:10.3390/cancers16101811)
Supplement: Supplementary file 1 [file cancers-16-01811-s001.zip › cancers-2968112-supplementary.pdf]

Supplementary Table S1. Treatment regimens for each cancer

Cancer (n)	Regimen	Number, %
Lung (384)	Pembrolizumab	133 (34.6)
	Nivolumab	104（27.1）
	Atezolizumab	92 (24.0)
	Ipilimumab	45 (11.8)
	Nivolumab + Ipilimumab	10 (2.6)
Digestive tract (216)	Nivolumab	190 (88.0)
	Pembrolizumab	14 (6.5)
	Nivolumab + Ipilimumab	8 (3.7)
	Atezolizumab	3 (1.4)
	Ipilimumab	1 (0.5)
Head and neck (162)	Nivolumab	111 (68.5)
	Pembrolizumab	50 (30.9)
	Nivolumab + Ipilimumab	1 (0.6)
Neuroepithelium (162)	Pembrolizumab	75 (46.3)
	Nivolumab	50 (30.9)
	Nivolumab + Ipilimumab	25 (15.4)
	Avelumab	10 (6.2)
	Atezolizumab	1 (0.6)
	Ipilimumab	1(0.6)
Melanoma (138)	Pembrolizumab	49 (35.5)
	Nivolumab	41 (29.7)
	Nivolumab + Ipilimumab	32 (22.2)
	Ipilimumab	16 (11.6)
Hepatobiliary (68)	Atezolizumab	64 (94.1)
	Pembrolizumab	2 (2.9)
	Nivolumab	2 (2.9)
Reproductive organ (64)	Pembrolizumab	37 (57.8)
	Atezolizumab	20 (31.3)
	Nivolumab	6 (9.4)
	Avelumab	1 (1.6)
Others (24)	Nivolumab	15 (62.5)
	Pembrolizumab	4 (16.7)
	Atezolizumab	3 (12.5)
	Avelumab	2 (8.3)
